# Development and psychometric validation of the hospitalized patients’ expectations for treatment scale**-**patient version

**DOI:** 10.3389/fpsyt.2023.1201707

**Published:** 2023-06-12

**Authors:** Chunfeng Xiao, Aoxue Wu, Yufei Wang, Tao Li, Yanping Duan, Yinan Jiang, Lili Shi, Xia Hong, Wenqi Geng, Jiarui Li, Jianhua Du, Jiaojiao Hu, Jinya Cao, Jing Wei

**Affiliations:** ^1^Department of Psychological Medicine, Peking Union Medical College Hospital, Chinese Academy of Medical Sciences and Peking Union Medical College, Beijing, China; ^2^Eight-Year Medical Doctor Program, Chinese Academy of Medical Sciences and Peking Union Medical College, Beijing, China; ^3^4+4 Medical Doctor Program, Chinese Academy of Medical Sciences and Peking Union Medical College, Beijing, China

**Keywords:** patient expectation, general hospital, doctor–patient relationship, outcome expectation, disease management expectancy, patient safety

## Abstract

**Objectives:**

A general expectation measurement of inpatients across wards is needed in the patient safety management systems of general hospitals. This study developed and psychometrically validated a new scale fulfilling the requirements above: the Hospitalized Patients’ Expectations for Treatment Scale-Patient version (HOPE-P).

**Methods:**

A total of 35 experts and ten inpatients were interviewed during the formulation of the HOPE-P scale, which was initially designed with three dimensions: doctor–patient communication expectations, treatment outcome expectations, and disease management expectancy. We recruited 210 inpatients from a general hospital in China and explored the reliability, validity, and psychometric characteristics of the questionnaire. Item analysis, construct validity, internal consistency and 7-day test–retest reliability analysis were applied.

**Results:**

Exploratory and confirmatory analyses supported a 2-dimension (doctor–patient communication expectation and treatment outcome expectation) structure with satisfactory model fit parameters (root mean square residual (RMR) = 0.035, a root-mean-square-error of approximation (RMSEA) = 0.072, comparative fit index (CFI) = 0.984, Tucker-Lewis index (TLI) = 0.970). Item analysis revealed an appropriate item design (r = 0.573–0.820). The scale exhibited good internal consistency, with Cronbach’s α of 0.893, 0.761, and 0.919 for the overall scale, the doctor–patient communication expectation subscale, and the treatment outcome expectation subscale, respectively. The 7-day test–retest reliability was 0.782 (*p* < .001).

**Conclusion:**

Our results indicated that the HOPE-P is a reliable and valid assessment tool to measure the expectations of general hospital inpatients, with a strong capacity to recognize patients’ expectations regarding doctor–patient communication and treatment outcomes.

## 1. Introduction

Patient expectations play a crucial role in the clinical context due to several reasons. Firstly, they serve as a valuable health economics indicator that can predict hospital length of stay (1). Secondly, they impact the outcomes of clinical trials, as the heterogeneity of patient preferences and expectations towards analgesics has revealed differentiated drug efficacy (2). Consequently, it is important to match patients’ expectations with the experimental therapeutics they receive. Furthermore, patient expectations have influenced the placebo effect in certain medical specialties. For example, in psychiatry, the response of patients with major depressive disorder (MDD) to escitalopram largely depends on their expectations (3), and evidence supports the role of expectations in subthalamic nucleus-deep brain stimulation (STN-DBS) for patients with Parkinson’s disease in the field of neurology (4).

Patient expectations are a significant factor that exhibits some correlation with various clinical variables. However, the strength and impact of this correlation differ depending on the disease, treatment, and patient population. Both doctors and patients primarily focus on treatment outcomes, and it has been found that expectations can predict the actual treatment outcome across a wide range of therapies. A meta-analysis, for instance, demonstrated a positive correlation between optimistic baseline expectations and improved post-treatment outcomes in psychotherapy (5). The role of expectations has also been observed in skin conditions such as allergic responses and inflammatory dermatoses (6), as well as in orthopedics (7). Positive expectations have been shown to predict better outcomes in psychotherapy (5), morphine analgesia (8), and total hip replacement (7). However, this positive correlation did not influence oral health-related quality of life in older edentulous patients following complete denture therapy (9), nor did it affect wrist function in conservatively treated radius fracture patients (10).

Patients’ expectations have various impacts on their safety. When patients have low treatment expectations, they may decline treatment (11), which subsequently affects their health. Unrealistic expectations of a “cure” are associated with unsatisfactory treatment adherence and poor illness control (12). Overestimating weight loss after bariatric surgery increases the risk of morbidity and mortality (13). Anticipated nausea prior to chemotherapy strongly predicts severe nausea afterwards (14), underscoring the significance of addressing expectations as an intervention target to protect patients from treatment-related adverse effects. Moreover, doctors’ decisions can be influenced by patients’ expectations, leading to improper prescriptions (15). Inaccurately high expectations of therapy are associated with lower post-surgery quality of life (16), directly compromising patient safety.

Consequently, there is a need for a comprehensive scale of patient expectations that can be applied across different diseases, treatments, and patient populations. In our previous research, we conducted six Delphi method seminars focused on patient safety, and experts widely recognized the importance and urgency of incorporating patient treatment expectations into the patient safety management system (17, 18). However, existing measures of patient expectations for inpatients in general hospitals are still inadequate. Most scales target a single treatment or disease (19), such as implantable cardioverter-defibrillator (ICD) (20), orthodontic (21), and gynecological treatment (22), making them incomparable among different hospital wards. Many scales also assess only specific aspects of treatment, such as expectations of physical functioning (23) or side effects (24), lacking a comprehensive and multidimensional approach. Furthermore, general and multidimensional instruments consist of numerous items (up to over 30) (25), making it challenging to assess patients’ expectations in the busy clinical environment.

To enhance the understanding of inpatients’ treatment expectations and improve patient safety management systems, we developed the hospitalized patients’ expectations for treatment scale-patient version (HOPE-P). The scale’s design principles include: (1) targeting hospitalized patients across different hospital wards; (2) incorporating factors concerning treatment expectations that are relevant to both patients and doctors; and (3) being concise and brief to facilitate practical use during the admissions process in clinical settings.

## 2. Materials and methods

### 2.1. Initial formulation of the scale

The opinions of 35 experts in psychiatry, surgery, internal medicine, nursing, and medical management were solicited. Ten inpatients who were undergoing treatment at Peking Union Medical College Hospital at the time were interviewed, including two cases of general surgery, one case of orthopedics, one case of vascular surgery, one case of plastic surgery, two cases of gastroenterology, one case of infectious medicine, and two cases of neurology. The Delphi method was used to pool the opinions of medical experts and patients. After three rounds of discussion, a common agreement was reached by all participants that not only medical treatment outcomes but also doctor–patient relationships and patients’ proper understanding of disease are important aspects that should be included during the evaluation of patient expectations. The final version of the HOPE-P was designed as a self-administered expectation measurement tool, designated generally for hospitalized patients of all specialties and indexed as nine items and three domains describing what patients expect from hospitalization. Two domains evaluated patient expectations, including expectations of doctor–patient communication (items 1–3) and treatment outcomes (items 4–8), with higher scores indicating higher expectations from doctors and hospitalization. One domain (item 9) evaluated patients’ cognition of disease management, which is a more realistic expectancy of the disease, and backward scoring was used for this item, with higher scores indicating higher expectations that future long-term treatment will not be needed after hospitalization. The reversal of the scoring of this item has the potential to reduce the likelihood of participants responding in a predictable or habitual manner. Each item is rated on a five-point Likert scale ranging from 1 to 5.

### 2.2. Participants

From February 2023 to March 2023, 210 inpatients were recruited from the gynecology, immunology, endocrinology, neurology, and cardiology wards of Peking Union Medical College Hospital, China. The inclusion criteria were age 10 or above and hospitalization for more than 24 h. The exclusion criteria were limited language skills, intellectual disability, visual or auditory impairment, and other factors hindering patients from providing informed consent or completing the scale. Informed consent was obtained from all participants. Especially for participants under 18 years old, additional informed consent of their parent or guardian was required. This study was approved by the Ethics Committee of the Peking Union Medical College Hospital, with assurance that data would be reported in aggregate form anonymously.

### 2.3. Scale administration

The scale (HOPE-P) was administered to patients within 24 h of ward admission. An applet on a smartphone developed and supported by the Department of Psychological Medicine, Peking Union Medical College Hospital served as a platform to complete the HOPE-P scale. Trained investigators informed the patients about the research and provided a QR code for the applet. Patients who agreed to participate scanned the QR code on their mobile phones, entered the main interface, and signed an informed consent form. For patients under 18 years of age, additional informed consent forms were authorized and signed by their parents or guardians. Following the instructions on the applet, and with the assistance of the investigator, patients were guided to provide sociodemographic information and complete the HOPE-P scale. The sociodemographic questionnaire collected information on age, sex, residence, marital status, family income, education level, employment status, etc. A total of 21 patients received a 7 days follow-up retest after the first completion of the HOPE-P scale.

### 2.4. Sample size calculation

The sample size calculation is based on the rules of thumb (or so-called blue-chips). Minimum sample sizes in absolute *N*s were the first rules of thumb, suggesting that any *N* > 200 offers adequate statistical power for data analysis (26). Besides, rules of thumbs also proposed that the ratio of the number of people (*N*) to the number of measured variables (*p*) should range from 5 with a minimum *N* > 100, to 10. A generally accepted ration is 10 cases per indicator variable. In light of the above considerations, the total sample size was determined at 200. The Kaiser–Meyer–Olkin (KMO) provided a measure of sampling adequacy.

### 2.5. Statistical analysis

Data analysis was conducted with IBM SPSS 26.0 and AMOS 26.0. The level of significance was established two-sided at a *p*-value of <0.05 throughout.

#### 2.5.1. Descriptive statistics

Sociodemographic characteristics and score distribution on the total scale and each subscale were described. Continuous variables are presented as mean ± standard deviation. Categorical variables were presented as numbers with percentages. Student’s *t*-test and one-way analysis of variance (ANOVA) with Scheffé’s post-hoc test (for three or more groups) were used as appropriate to detect any differences in the scores concerning sociodemographic factors.

#### 2.5.2. Item analysis

The total score of the scale ranged from high to low, with the scores in the top 27% and the bottom 27% being the high- and low-score groups, respectively. The critical ratio (CR) value was obtained using a *t*-test to compare the differences between the high- and low-scoring groups. Correlation analysis was performed between the scores of each item and the total score minus the item score, with a significant correlation coefficient larger than 0.4 demonstrating the item has an appropriate design.

#### 2.5.3. Structural validity

To investigate the unexplored structure of the HOPE-P scale, exploratory factor analysis (EFA) was performed on a random half-sample of the collected data using IBM SPSS 26.0. The Kaiser–Meyer–Olkin (KMO) measure of sample adequacy and the Bartlett’s test of sphericity were conducted in priori to check data suitability and sampling adequacy. KMO values greater than 0.70 and a *p*-value <0.001 of Bartlett’s test were regarded as indicating a sufficient correlation between the items to suit structure detection. Varimax rotation was chosen, and factors with eigenvalues greater than 1 were extracted. A total factor loading of >60% was considered acceptable.

To examine the structure obtained in the EFA and the primarily designed structure, confirmatory factor analysis (CFA) was conducted on the other half of the sample using AMOS 26.0. An RMR <0.05, RMSEA value ≤0.10, with CFI, normed fit index (NFI), non-normed fit index (NNFI), incremental fit index (IFI), Tucker–Lewis index (TFI), and goodness of fit index values >0.9 were used to identify appropriate global model fits.

#### 2.5.4. Reliability analysis

To test the internal consistency of the newly developed instrument, Cronbach’s *α* coefficients and McDonald’s *ω* coefficients were computed for the entire scale and its subscales, and 95% confidence intervals (95% CI) are provided. A Cronbach’s *α* coefficient/McDonald’s *ω* coefficient above 0.7 indicates high internal consistency, while a value above 0.9 implies redundancy. The Pearson correlation coefficient or Spearman correlation coefficient between the first test and the retest was calculated to access the 7 days test–retest reliability as appropriate based on the results of Kolmogorov–Smirnov test.

## 3. Results

### 3.1. Sociodemographic characteristics and score distribution

Table 1 presents the sociodemographic characteristics and score distributions of the entire scale and each subscale. A total of 210 patients participated in the study, with an average age of 42.72 ± 14.60, 146 (69.5%) of whom were female. The average total score of the HOPE-P scale was 38.70 ± 4.23 (full score = 45). The average score of each subscale was as follows: 13.02 ± 2.17 for doctor–patient communication expectation subscale (subscale A, full score = 15), 23.55 ± 2.91 for treatment outcome expectation subscale (subscale B, full score = 25), and 2.12 ± 1.37 for disease management expectancy subscale (subscale C, full = score 5).

**Table 1:** Sociodemographic characteristics and score distribution of the total sample.

Variables	Number (%)	Overall scale (mean ± SD)	Subscale A (doctor–patient communication expectation) (mean ± SD)	Subscale B (treatment outcome expectation) (mean ± SD)	Subscale C (disease management expectancy) (mean ± SD)
**Age**	*N* = 210	*p* = 0.468	*p* = 0.135	*p* = 0.525	*p* = 0.134
10–20	11 (5.2%)	37.27 ± 6.44	12.18 ± 3.19	22.73 ± 4.45	2.36 ± 1.69
21–40	88 (41.9%)	38.47 ± 4.91	12.74 ± 2.24	23.38 ± 3.42	2.35 ± 1.40
41–60	82 (39.0%)	39.17 ± 3.37	13.23 ± 2.08	23.94 ± 2.01	2.00 ± 1.32
61–80	27 (12.9%)	38.37 ± 3.04	13.52 ± 1.58	23.22 ± 2.78	1.63 ± 1.18
>80	2 (1.0%)	41.50 ± 0.71	15.00 ± 0.00	24.50 ± 0.71	2.00 ± 1.41
**Gender**	*N* = 210	*p* = 0.115	*p* = 0.284	*p* = 0.131	*p* = 0.967
Male	64 (30.5%)	38.00 ± 5.26	12.78 ± 2.68	23.09 ± 3.76	2.12 ± 1.46
Female	146 (69.5%)	39.00 ± 3.67	13.13 ± 1.92	23.75 ± 2.43	2.12 ± 1.33
**Residence**	*N* = 209	*p* = 0.062	*p* = 0.062	*p* = 0.272	*p* = 0.657
Urban	177 (84.7%)	38.49 ± 4.41	12.91 ± 2.27	23.48 ± 3.05	2.10 ± 1.34
Rural	32 (15.3%)	40.00 ± 2.66	13.69 ± 1.38	24.09 ± 1.80	2.22 ± 1.54
**Marital status**	*N* = 209	<0.001^***^	*p* = 0.009^**^	*p* = 0.001^**^	*p* = 0.084
Single	37 (17.7%)	38.41 ± 4.20^##^	12.59 ± 2.23	23.51 ± 2.84^##^	2.30 ± 1.29
Married	153 (73.2%)	39.02 ± 3.25^###^	13.25 ± 1.86^#^	23.74 ± 2.19^##^	2.03 ± 1.31
Divorced	9 (4.3%)	40.22 ± 2.95^##^	12.89 ± 1.97	24.11 ± 1.62^#^	3.22 ± 1.92
Widowed	3 (1.4%)	39.33 ± 2.89	13.33 ± 2.89	24.67 ± 0.58	1.33 ± 0.58
Other	7 (3.3%)	31.71 ± 12.85	10.43 ± 5.29	19.14 ± 9.67	2.14 ± 1.95
**Monthly family income**	*N* = 209	*p* = 0.109	*p* = 0.014^*^	*p* = 0.024^*^	*p* = 0.002^**^
<4,000 RMB	27 (12.9%)	37.30 ± 8.26	12.22 ± 3.47	22.19 ± 5.79	2.89 ± 1.67
4,000–8,000 RMB	57 (27.3%)	39.37 ± 3.24	13.63 ± 2.14^^^	23.95 ± 2.29^^^	1.79 ± 1.16^^^^
>8,000 RMB	125 (59.8%)	38.74 ± 3.18	12.93 ± 1.74	23.70 ± 2.05^^^	2.10 ± 1.33^^^
**Employment status**	*N* = 209	*p* = 0.410	*p* = 0.467	*p* = 0.325	*p* = 0.249
Student	15 (7.2%)	37.40 ± 5.58	12.27 ± 2.76	22.67 ± 3.87	2.47 ± 1.60
Employed	109 (52.2%)	39.24 ± 3.03	13.19 ± 1.73	23.95 ± 1.95	2.09 ± 1.30
Unemployed	27 (12.9%)	38.26 ± 5.81	12.96 ± 2.68	23.04 ± 4.08	2.26 ± 1.43
Retired	38 (18.2%)	38.32 ± 3.76	13.16 ± 2.31	23.39 ± 2.64	1.76 ± 1.20
Other	20 (9.6%)	38.30 ± 6.50	12.55 ± 2.83	23.25 ± 4.59	2.50 ± 1.70
**Educational level**	*N* = 209	*p* = 0.07	*p* = 0.210	*p* = 0.017^*^	*p* = 0.437
Elementary	7 (3.3%)	40.71 ± 1.25	14.43 ± 1.13	25.00 ± 0.00	1.29 ± 0.76
Junior	17 (8.1%)	36.71 ± 8.08	12.94 ± 3.58	21.65 ± 5.62	2.12 ± 1.42
High school	50 (23.9%)	38.22 ± 5.08	12.66 ± 2.67	23.44 ± 3.57	2.12 ± 1.62
College or higher	135 (64.6%)	39.06 ± 3.06	13.10 ± 1.73	23.79 ± 2.00^ϕ^	2.16 ± 1.28
**Wards**	*N* = 210	*p* = 0.322	*p* = 0.771	*p* = 0.314	*p* = 0.637
Gynecology	99 (47.1%)	38.80 ± 4.15	12.89 ± 2.06	23.66 ± 2.69	2.25 ± 1.31
Immunology	1 (0.5%)	33.00 ± 0.00	12.00 ± 0.00	19.00 ± 0.00	2.00 ± 0.00
Endocrinology	33 (15.7%)	39.30 ± 4.08	13.36 ± 2.22	23.85 ± 2.80	2.09 ± 1.47
Neurology	22 (10.5%)	39.77 ± 1.90	13.64 ± 1.65	24.64 ± 0.85	1.50 ± 1.10
Cardiology	55 (26.2%)	37.82 ± 4.95	12.84 ± 2.50	22.84 ± 3.65	2.15 ± 1.47

Student’s *t*-test and one-way ANOVA with Scheffé’s post-hoc test (for three or more groups) were used as appropriate. ^*^p < 0.05. Compared with marital status = other: ^#^*p* < 0.05, ^##^*p* < 0.01, ^###^*p* < 0.001. Compared with monthly family income = <4,000 RMB: ^^^*p* < 0.05, ^^^^*p* < 0.01, ^^^^^*p* < 0.001. Compared with educational level = junior: ^ϕ^*p* < 0.05, ^ϕϕ^*p* < 0.01, ^ϕϕϕ^*p* < 0.001.

The statistical analysis revealed no significant differences in HOPE-P ratings based on gender, age, residence, or employment status. However, there were significant differences among patients of different marital status in the total score of HOPE-P [*F*_(4, 204)_ = 5.879, *p* < 0.001], subscale A [*F*_(4, 204)_ = 3.472, *p* = 0.009], and subscale B [*F*_(4, 204)_ = 4.711, *p* = 0.001]. Inpatients who were categorized as “other” marital status had lower scores compared to single (31.71 ± 12.85 vs. 38.41 ± 4.20, *p* = 0.03), married (31.71 ± 12.85 vs. 39.02 ± 3.25, *p* < 0.001), and divorced inpatients. Inpatients of “other” marital status also had lower subscale A scores compared to married inpatients (10.43 ± 5.29 vs. 13.25 ± 1.86, *p* = 0.021). Similarly, inpatients of “other” marital status showed lower scores in subscale B compared to single (19.14 ± 9.67 vs. 23.51 ± 2.84, *p* = 0.008), married (19.14 ± 9.67 vs. 23.74 ± 2.19, *p* = 0.002), and divorced inpatients (19.14 ± 9.67 vs. 24.11 ± 1.62, *p* = 0.017). Regarding monthly family income levels, significant differences were found in subscale A [*F*_(4, 206)_ = 4.323, *p* = 0.014], subscale B [*F*_(4, 206)_ = 3.794, *p* = 0.024], and subscale C [*F*_(4, 206)_ = 6.223, *p* = 0.002]. Inpatients with a monthly family income of less than 4,000 had lower scores in subscale A compared to those with a monthly family income of 4,000–8,000 (12.22 ± 3.47 vs. 13.63 ± 2.14, *p* = 0.02). Inpatients with a monthly family income of less than 4,000 also had lower scores in HOPE-P subscale B compared to those with a monthly family income of 4,000–8,000 (22.19 ± 5.79 vs. 23.95 ± 2.29, *p* = 0.033) and more than 8,000 (22.19 ± 5.79 vs. 23.70 ± 2.05, *p* = 0.046). Additionally, inpatients with a monthly family income of less than 4,000 had lower scores in HOPE-P subscale C compared to those with a monthly family income of 4,000–8,000 (2.89 ± 1.67 vs. 1.79 ± 1.16, *p* = 0.002) and more than 8,000 (2.89 ± 1.67 vs. 2.10 ± 1.33, *p* = 0.023). Furthermore, significant differences were found among patients of different educational levels in subscale B [*F*_(3, 205)_ = 3.478, *p* = 0.017]. Inpatients with a junior educational level had lower scores in subscale B compared to those with a college or higher educational level (21.65 ± 5.62 vs. 23.79 ± 2.00, *p* = 0.038).

### 3.2. Item analysis

The CR values of items 1–8 ranged from 6.036 to 8.354, and the differences between the low- and high-scoring groups were all significant. The scores for items 1–8 and the total score were all significantly correlated, with coefficients between 0.573 and 0.820, all of which hare above 0.40. Item 9 had the lowest CR value (0.071) and a significant negative correlation with the total score. Table 2 presents the results of the item analysis.

**Table 2:** Item analysis.

	Item	Critical ration (CR value)	Corrected item-total correlation
Subscale A: doctor–patient communication expectation	Q1. The doctor listens to my opinions on treatment	8.169^**^	0.573^**^
	Q2. During this hospitalization, the doctor fully explains the state of illness to me and negotiates medical decisions with me	8.341^**^	0.809^**^
	Q3. During this hospitalization, the doctor is caring	8.354^**^	0.723^**^
Subscale B: treatment outcome expectation	Q4. Through this hospitalization, the disease can be definitely diagnosed	7.960^**^	0.820^**^
	Q5. Through this hospitalization, symptoms can be improved	6.801^**^	0.808^**^
	Q6. Through this hospitalization, the disease can be cured	7.560^**^	0.764^**^
	Q7. Through this hospitalization, I can restore work/family functions	7.117^**^	0.763^**^
	Q8. Through this hospitalization, I can take care of myself	6.036^**^	0.721^**^
Subscale C: disease management expectancy	Q9. After this hospitalization, I need to maintain long-term treatment	0.071	−0.096

^*^p < 0.05, ^**^p < 0.01.

### 3.3. Structural validity

Based on the results of the item analysis and considering the initial design concept of the scale, we conducted an EFA on eight items (except item 9) and nine items in a random half-sample (*N* = 105). Factorability of the inter-correlation matrix of the eight items and nine items were confirmed by the KMO values of 0.871 and 0.872, respectively, and the Bartlett’s sphericity test (eight items: *χ*^2^ = 706.145, d*f* = 28, *p* < 0.001; nine items: *χ*^2^ = 752.300, d*f* = 36, *p* < 0.001). The initial solutions of eight and nine items revealed two factors with eigenvalues above 1, cumulatively accounting for 80.146 and 74.445% of the variation, respectively, which can be regarded as acceptable. Table 3 lists the item loading values.

**Table 3:** Factor Analysis.

	Item	EFA (8 items)			EFA (9 items)		
		Loadings on factor 1	Loadings on factor 2	Community	Loadings on factor 1	Loadings on factor 2	Community
Subscale A: doctor–patient communication expectation	Q1	0.063	0.875	0.770	0.056	0.905	0.823
	Q2	0.475	0.728	0.755	0.504	0.660	0.690
	Q3	0.345	0.840	0.825	0.368	0.797	0.770
Subscale B: treatment outcome expectation	Q4	0.705	0.536	0.785	0.726	0.490	0.767
	Q5	0.824	0.354	0.805	0.829	0.339	0.803
	Q6	0.827	0.215	0.730	0.838	0.185	0.736
	Q7	0.904	0.201	0.859	0.891	0.232	0.847
	Q8	0.920	0.191	0.884	0.906	0.226	0.871
Subscale C: disease management expectancy	Q9	/	/	/	−0.238	−0.581	0.394

EFA, exploratory factor analysis; CFA, confirmatory factor analysis; factor 1, treatment outcome expectations; factor 2, doctor–patient communication expectations.

Based on the EFA results, combined with our primary design principles, we classified items 1–3 into one dimension—doctor–patient communication expectations—and measured patients’ expectations of doctor–patient communication. Items 4–8 were categorized into one dimension—treatment outcome expectations—to measure patients’ expectations regarding the treatment outcome of the disease. Although including item 9 as a separate dimension in the scale may not be entirely appropriate based on the results of the EFA, we acknowledge the importance of item 9’s investigation of disease management expectancy in comprehensively assessing hospitalized patients’ expectations for treatment. Therefore, we considered item 9 a separate dimension and validated it using CFA. It thus formed a single dimension, namely disease management expectancy and measuring patients’ expectations for the long-term management of the disease. The three-dimension structure was initially aligned with the design purpose.

**Figure 1:**
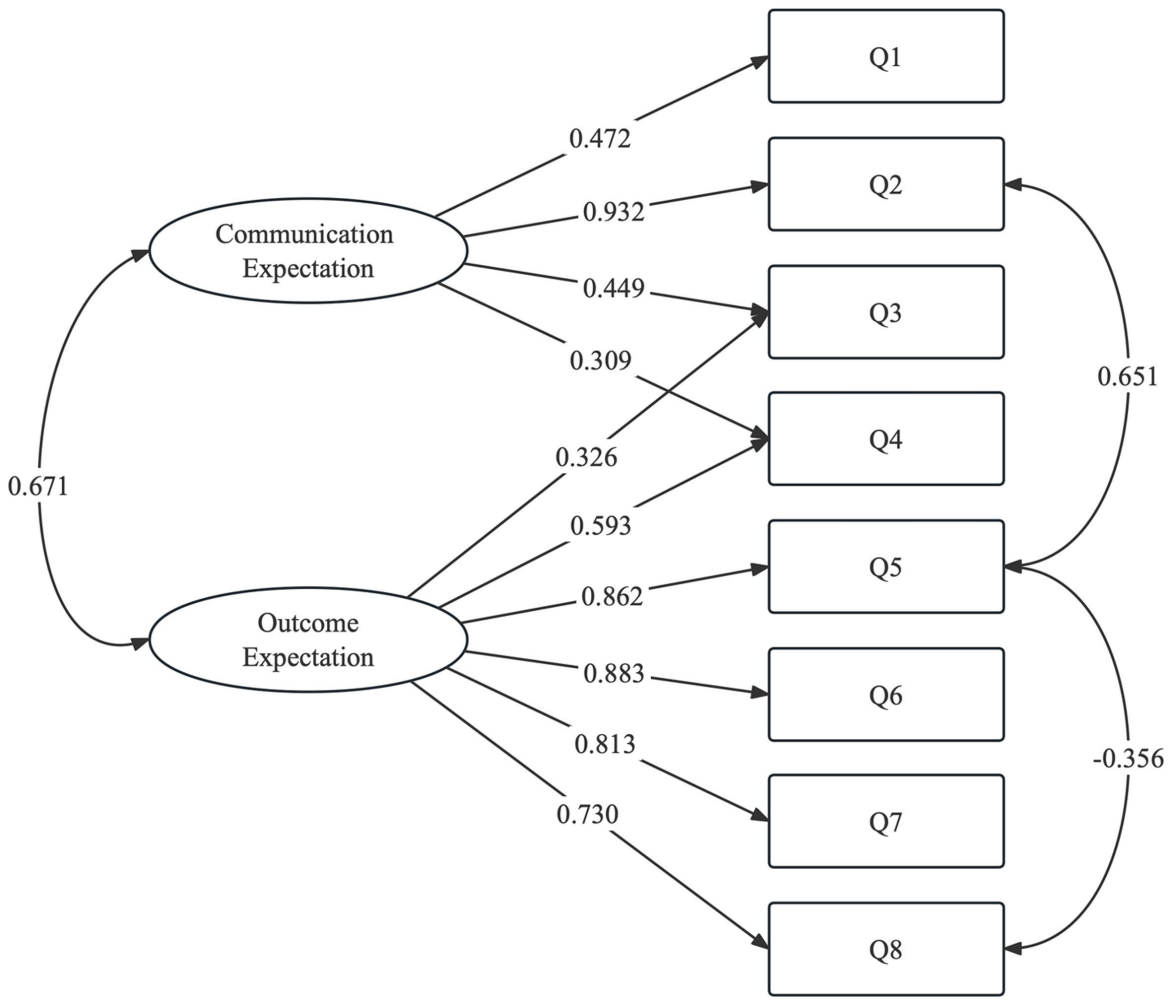
Factor structure of hospitalized patients’ expectations for treatment scale-patient version (HOPE-P), based on an eight-item two-factor model (modified).

CFA was conducted on the other half of the sample (*N* = 105). We tested several CFA models, of which the eight-item two-factor (modified) model was considered the best match with satisfactory model fit indices of RMR = 0.035, RMSEA = 0.072, CFI = 0.984, and TLI = 0.970. Figure 1 shows the factor loadings of each item in the model, and Table 4 presents the results of the CFA.

**Table 4:** Results of CFA models.

CFA models	*χ* ^2^	d*f*	*p*	RMSEA	SRMR	CFI	TLI	Δ*χ*^2^
Nine-item one-factor model	145.504	27	<0.001	0.204	0.093	0.819	0.759	
Nine-item three-factor model (modified)	85.791	22	<0.001	0.166	0.092	0.915	0.861	59.713
Eight-item one-factor model	69.939	20	<0.001	0.154	0.059	0.901	0.861	
Eight-item two-factor model (unmodified)	56.983	19	<0.001	0.138	0.052	0.924	0.889	12.956
Eight-item two-factor model (modified)	23.133	15	0.0814	0.072	0.035	0.984	0.970	46.806

### 3.4. Reliability analysis

A reliability analysis based on an eight-item two-factor model (modified) revealed satisfactory reliability of the instrument. Internal consistency of developed instrument was good, for the overall scale [Cronbach’s *α* = 0.893, 95% CI = (0.869, 0.913)], subscale A [Cronbach’s *α* = 0.761, 95% CI = (0.699, 0.812)], and subscale B [Cronbach’s *α* = 0.919, 95% CI = (0.900, 0.935)]. McDonald’s *ω* coefficients of the HOPE-P full scale, subscale A and B were 0.890 [95% CI = (0.867, 0.913)], 0.761 [95% CI = (0.705, 0.817)], and 0.921 [95% CI = (0.904, 0.938)], respectively. The 7 days test–retest reliability of the overall scale was 0.670 (*p* = 0.001).

## 4. Discussion

In this study, 210 inpatients from a general hospital in China were recruited to explore the reliability, validity, and psychometric characteristics of the HOPE-P, a new scale consisting of three subscales measuring the overall expectations of hospitalized patients in a general hospital in a multidimensional manner. With an initial design of three dimensions with nine items in total covering doctor–patient communication, treatment outcome expectations, and disease management expectancy, this scale provides a feasible and convenient approach for evaluating hospitalized patients’ expectations for treatment in Chinese culture. The results of this study support the good reliability of the overall scale, as well as the satisfactory reliability and validity of the first two subscales.

Expectation-focused psychological interventions (EFPIs) have been shown to positively affect clinical outcomes in various medical conditions, including cancer, chronic pain, and coronary heart disease (27). These interventions are based on the understanding that expectation management plays a crucial role in treatment effectiveness. Previous studies have proposed that the three pillars of expectation management are trust, communication, and patient education (28). These pillars align with our three subscales, in which trust and communication correspond to the communication subscale and patient education can help build appropriate cognition about treatment outcomes and disease management expectancy.

Specifically, the first subscale focused on doctor–patient communication expectations, consisting of three items. These items assessed the extent to which patients’ treatment opinions were listened to, the doctor effectively informed patients about the disease condition and engaged in collaborative medical decision-making, and the doctor demonstrated a caring attitude. This design aligns with key concepts in high-quality doctor–patient communication, including patient autonomy, shared decision-making, and humanitarianism (29, 30). The second subscale addressed treatment outcome expectations and consisted of five items. These items measured patients’ expectations regarding treatment outcome-related factors such as disease diagnosis, symptom improvement, and cure, as well as functional outcomes, particularly the restoration of social function. These items reflected patients’ beliefs about the potential health consequences of treatment. It is worth noting that treatment outcome expectations are influenced by doctor–patient communication. For instance, in the context of cognitive-behavioral therapy (CBT) for patients with generalized anxiety disorder (GAD), higher outcome expectations were associated with a stronger therapeutic alliance between the therapist and patient in subsequent sessions (31).

Disease management expectancy is a single-item subscale that reflects patients’ cognition of the disease’s long-term management. Expectancies differ from expectations: the former is based more on cognition than on values; thus, it should be more realistic and scientific than the latter. In this study, we did not observe any significant differences in the scores of the three dimensions among the clinical departments, which could be attributed to the fact that the participants included in our study were all from departments specializing in chronic diseases. Consistent with the clinical reality, patients with chronic diseases have similar expectations regarding the main aspects of the diagnosis and treatment process and expectancy regarding the prognosis of the disease. Furthermore, we found that doctor–patient communication and treatment outcome expectations were both negatively correlated with disease management expectancy (Spearmann’s *r* = −0.346, *p* < 0.001; Spearmann’s *r* = −0.318, *p* < 0.001, respectively) (see Tables 1, 2 in Supplement 1). A high expectancy score was significantly correlated with a low expectation score. A possible mechanism may be the competing roles of reality and desired outcome on what patients expect. When a patient is more realistic, their wish-based expectation score declines.

It should be noted that our study did not support the inclusion of the disease management expectancy subscale in the HOPE-P. This could be due to several factors, including potential issues with the design of the item or its standalone nature. Based on our results, we compared multiple CFA models and selected an eight-item, two-factor model for further analysis. However, attention to disease management expectancy remains important for a comprehensive evaluation of the treatment expectations of hospitalized patients, with the aim of improving personalized medical care. CFA revealed that items 3 and 4 loaded onto both factors. Furthermore, there was a correlation between items 2 and 5 and between items 5 and 8, suggesting that shared decision-making between doctors and patients may influence patients’ treatment outcome expectations. Future research could use structural equation modeling (SEM) or cross-lagged analysis to explore causality and investigate the feasibility of improving patients’ treatment outcome expectations by promoting shared decision-making.

The average HOPE-P score exceeded 85% of the maximum score (38.7/45; 86%), indicating high treatment expectations. Unrealistic treatment expectations have been found across various medical fields, reflecting patients’ tendencies to overestimate the effects/outcomes of their therapy. Moreover, this study indicates possible factors that might influence patients’ expectations of treatment, such as unsatisfactory marital relationships (e.g., separated, divorced), low monthly family income, and low educational levels.

Our study has several limitations. First, the validation was conducted mainly in five wards of the hospital, thus limiting the representativeness of the participants. Because this was only a pilot validation of the tool, future studies should be conducted in more wards to further test its application in other medical fields. Second, suspicious ceiling effects were observed in patient rating scores. We analyzed the possibility of unrealistic expectations from patients, which is expected to explain this overestimation. Doctors’ ratings of what can actually be achieved during hospitalization using a matching scale are a promising way to answer this question and deserve further investigation.

The value of this scale for patient safety management systems in general hospitals and other potential clinical applications is also worth exploring.

## Data availability statement

The raw data supporting the conclusions of this article will be made available by the authors, without undue reservation.

## Ethics statement

The studies involving human participants were reviewed and approved by Ethics Committee of Peking Union Medical College Hospital. Written informed consent to participate in this study was provided by the participants’ legal guardian/next of kin.

## Author contributions

CX and AW wrote the manuscript. YW revised the manuscript as needed. CX, YW, and AW contributed to statistical analysis and data interpretation. JC and JW contributed to the initial conceptualization of the scale and proposed the critical design of the study. JC, JW, YW, TL, YD, YJ, LS, XH, WG, JL, JD, JH, AW, and CX contributed significantly to scale revision and data collection. All authors contributed to the article and approved the submitted version.

## Funding

This research was funded by the Capital Fund for Health Improvement and Research (2022-2-4012), the National High-Level Hospital Clinical Research Fund (2022-PUMCH-B-093), and Education Fund for the Reform and Construction of Comprehensive Evaluation and Assessment System in Clinical Medicine (X226105).

## Conflict of interest

The authors declare that the research was conducted in the absence of any commercial or financial relationships that could be construed as a potential conflict of interest.

## Publisher’s note

All claims expressed in this article are solely those of the authors and do not necessarily represent those of their affiliated organizations, or those of the publisher, the editors and the reviewers. Any product that may be evaluated in this article, or claim that may be made by its manufacturer, is not guaranteed or endorsed by the publisher.
