# Supplementary material for: Development and psychometric validation of the hospitalized patients’ expectations for treatment scale-patient version
Source: Front Psychiatry. 2023 Jun 12;14:1201707. doi: 10.3389/fpsyt.2023.1201707 (PMC10291120; doi:10.3389/fpsyt.2023.1201707)
Supplement: Supplementary file 1 [file Table_1.docx]

**eTable 1.** Correlations between items of HOPE-P.

|  | Q1 | Q2 | Q3 | Q4 | Q5 | Q6 | Q7 | Q8 | Q9 |
| --- | --- | --- | --- | --- | --- | --- | --- | --- | --- |
| Q1 | 1.000 | 0.516^**^ | 0.463^**^ | 0.318^**^ | 0.293^**^ | 0.183^**^ | 0.227^**^ | 0.244^**^ | -0.278^**^ |
| Q2 |  | 1.000 | 0.544^**^ | 0.552^**^ | 0.481^**^ | 0.382^**^ | 0.395^**^ | 0.349^**^ | -0.173^*^ |
| Q3 |  |  | 1.000 | 0.589^**^ | 0.405^**^ | 0.402^**^ | 0.381^**^ | 0.351^**^ | -0.366^**^ |
| Q4 |  |  |  | 1.000 | 0.632^**^ | 0.554^**^ | 0.530^**^ | 0.561^**^ | -0.291^**^ |
| Q5 |  |  |  |  | 1.000 | 0.579^**^ | 0.657^**^ | 0.619^**^ | -0.251^**^ |
| Q6 |  |  |  |  |  | 1.000 | 0.620^**^ | 0.603^**^ | -0.245^**^ |
| Q7 |  |  |  |  |  |  | 1.000 | 0.757^**^ | -0.273^**^ |
| Q8 |  |  |  |  |  |  |  | 1.000 | -0.321^**^ |
| Q9 |  |  |  |  |  |  |  |  | 1.000 |

^*^*P*< 0.05, ^**^*P*< 0.01

**eTable 2.** Correlations between domains of HOPE-P.

|  | D1 | D2 | D3 |
| --- | --- | --- | --- |
| D1 | 1.000 | 0.502^**^ | -0.346^**^ |
| D2 |  | 1.000 | -0.318^**^ |
| D3 |  |  | 1.000 |

D1: domain 1, doctor-patient communication expectation; D2: domain 2, treatment outcome expectation; D3: domain 3, disease management expectancy.

^*^*P*< 0.05, ^**^*P*< 0.01

**eTable 3.** The internal consistency of the HOPE-P as assessed by the Cronbach’s α and McDonald's ω coefficient.

| Item | Mean | Standard deviation | CITC | Cronbach’s α if item deleted | McDonald's ω if item deleted |
| --- | --- | --- | --- | --- | --- |
| Overall Scale | | | | |  |
| Q1 | 4 | 1.083 | 0.436 | 0.918 | 0.918 |
| Q2 | 4.57 | 0.737 | 0.75 | 0.872 | 0.870 |
| Q3 | 4.45 | 0.776 | 0.688 | 0.877 | 0.876 |
| Q4 | 4.69 | 0.646 | 0.798 | 0.869 | 0.862 |
| Q5 | 4.76 | 0.597 | 0.789 | 0.872 | 0.868 |
| Q6 | 4.7 | 0.627 | 0.727 | 0.876 | 0.873 |
| Q7 | 4.69 | 0.716 | 0.734 | 0.873 | 0.871 |
| Q8 | 4.72 | 0.751 | 0.691 | 0.877 | 0.874 |
| Subscale A: Doctor–patient Communication Expectation | | | | |  |
| Q1 | 4 | 1.083 | 0.54 | 0.803 | N/A |
| Q2 | 4.57 | 0.737 | 0.676 | 0.616 | N/A |
| Q3 | 4.45 | 0.776 | 0.63 | 0.651 | N/A |
| Subscale B: Treatment Outcome Expectation | | | | |  |
| Q4 | 4.69 | 0.646 | 0.756 | 0.907 | 0.912 |
| Q5 | 4.76 | 0.597 | 0.807 | 0.899 | 0.903 |
| Q6 | 4.7 | 0.627 | 0.796 | 0.9 | 0.904 |
| Q7 | 4.69 | 0.716 | 0.83 | 0.892 | 0.893 |
| Q8 | 4.72 | 0.751 | 0.784 | 0.904 | 0.905 |

CITC: Corrected Item-Total Correlation

N/A: Not Available

The analysis was performed based on an 8-item 2-factor model (modified)
